# Comparative Analysis of the Effects of Maternal Hypoxia and Placental Ischemia on HIF1-Dependent Metabolism and the Glucocorticoid System in the Embryonic and Newborn Rat Brain

**DOI:** 10.3390/ijms252413342

**Published:** 2024-12-12

**Authors:** Oleg Vetrovoy, Sofiya Potapova, Viktor Stratilov, Ekaterina Tyulkova

**Affiliations:** Laboratory of Regulation of Brain Neuronal Functions, Pavlov Institute of Physiology, Russian Academy of Sciences, Makarova emb. 6, 199034 Saint-Petersburg, Russiastratilov.v@icloud.com (V.S.);

**Keywords:** maternal hypoxia, placental ischemia, glucocorticoid system, HIF1, glycolysis, pentose phosphate pathway

## Abstract

Prenatal hypoxia, often accompanied by maternal glucocorticoid stress, can predispose offspring to neurological disorders in adulthood. If placental ischemia (PI) primarily reduces fetal oxygen supply, the maternal hypoxia (MH) model also elicits a pronounced fetal glucocorticoid exposure. Here, we compared MH and PI in rats to distinguish their unique and overlapping effects on embryonic and newborn brain development. We analyzed glucocorticoid transport into the developing brain, glucocorticoid receptor (GR) expression, and GR-dependent transcription, along with key enzymes regulating glucocorticoid metabolism in maternal (MP) and fetal placentas (FP) and in the brain. Additionally, we examined hypoxia-inducible factor 1-alpha (HIF1α) and its downstream genes, as well as glycolysis and the pentose phosphate pathway, both associated with the transport of substrates essential for glucocorticoid synthesis and degradation. Both MH and PI induced HIF1-dependent metabolic alterations, enhancing glycolysis and transiently disrupting redox homeostasis. However, only MH caused a maternal glucocorticoid surge that altered early fetal brain glucocorticoid responsiveness. Over time, these differences may lead to distinct long-term outcomes in neuronal structure and function. This work clarifies the individual contributions of hypoxic and glucocorticoid stresses to fetal brain development, suggesting that combining the MH and PI models could provide valuable insights for future investigations into the mechanisms underlying developmental brain pathologies, including non-heritable psychoneurological and neurodegenerative disorders.

## 1. Introduction

Oxygen insufficiency poses serious risks at all life stages, severely compromising tissue structure and function in both adults and developing organisms [1,2,3]. Notably, the most critical pathologies linked to hypoxic episodes include strokes and cardiovascular diseases [4,5]. Moreover, oxygen insufficiency, being part of various detrimental factors, is particularly perilous during intrauterine development and the early postnatal period. It is not only a leading cause of intrauterine fetal demise but also contributes to significant malfunctions in the brain during later postnatal development [6,7], primarily due to profound epigenetic modifications that program various brain systems, resulting in persistent effects throughout life [8,9,10]. Consequently, episodes of prenatal hypoxia at various stages of prenatal development can contribute to the onset of schizophrenia, depressive-like disorders, addictions, autism spectrum disorders, and neurodegenerative diseases [11,12,13].

In addition to the direct effects of prenatal hypoxia, which include metabolic pathway shifts and oxidative stress [14,15] leading to intrauterine growth restriction [16], prenatal hypoxia is often associated with the maternal glucocorticoid system’s response to hypoxic episodes [17]. This condition is widely considered comorbid with various prenatal pathologies, including placental ischemia and preeclampsia [18,19]; maternal infections, such as chorioamnionitis; and maternal vascular diseases, including chronic hypertension and diabetes, which are associated with compromised placental blood flow [13]. However, investigating the long-term consequences of fetal hypoxia presents challenges in discerning which of these factors exerts the more significant impact.

Understanding the common principles of brain development in humans and rodents facilitates the modeling of prenatal developmental pathologies in vivo [7]. To investigate the molecular, cellular, and epigenetic mechanisms underlying disturbances caused by adverse effects on the fetus during critical developmental periods, it is essential to employ diverse experimental approaches enabling the examination of various elements of pathological conditions using model organisms [20]. However, to validate the use of these experimental approaches for investigating the mechanisms underlying developmental pathologies, a detailed characterization of the molecular and cellular events they induce is required. In this study, we utilized two models—direct fetal hypoxia via intrauterine ischemia (placental ischemia, PI) [21] and maternal stress response to hypoxia (maternal hypoxia, MH) [11,22,23] to characterize their similarities and specific effects on brain development.

We characterized the influence of MH and PI on the accumulation of hypoxia-inducible factor 1 (HIF1) protein, its mRNA expression, and the expression of glucocorticoid receptor (GR) protein. Additionally, we examined GR-dependent expression [24] and the expression of enzymes involved in glucocorticoid metabolism, specifically 11beta-hydroxysteroid dehydrogenase 1 (HSD11B1) and 11beta-hydroxysteroid dehydrogenase 2 (HSD11B2) [25], in both the maternal and fetal parts of the placenta (MP and FP, respectively) one day after the onset of hypoxic episodes and before parturition (e15 and e20, respectively).

To investigate the effects of MH and PI on the glucocorticoid system in the developing brain of offspring, we assessed corticosterone levels, mRNA expression, and protein levels of GR. We also examined GR-dependent gene expression (*zbtb16*, *fkbp5*, and *dusp1*) and the expression of glucocorticoid metabolism enzymes HSD11B1 and HSD11B2 during prenatal (e15, e16, e17, e20) and early postnatal ontogenesis (p1). To evaluate the effects of MH and PI on HIF1-dependent metabolism during these periods, we analyzed HIF1α mRNA expression and HIF-dependent gene expression (*glut1*, *hk1*, *pfkb3*, *ldha*, *pdk1*, *mct4*), as well as the functional consequences on anaerobic glycolysis (protein content, lactate dehydrogenase A activity (LDHA), and lactate and pyruvate concentrations) and the pentose phosphate pathway (activity of glucose-6-phosphate dehydrogenase, NADPH concentration). Additionally, we assessed redox parameters mediated by the balance between glycolysis and the pentose phosphate pathway, including reduced glutathione concentration (GSHred) and malondialdehyde (MDA) levels.

## 2. Results

### 2.1. Expression of HIF1α and Glucocorticoid Receptors, Glucocorticoid-Dependent Transcription, and Corticosterone Metabolism in Maternal and Fetal Placenta During MH or PI

**Figure 1:**
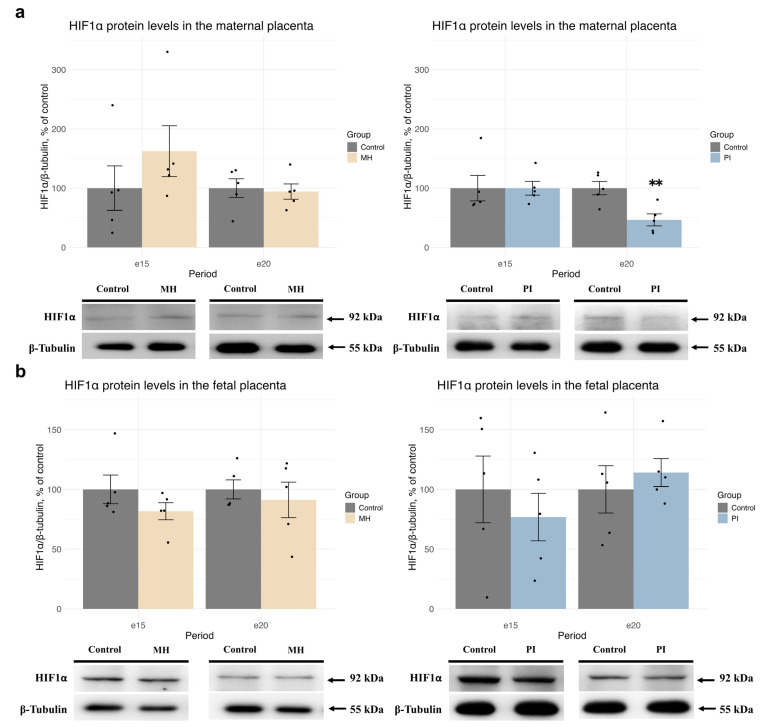
The effect of MH and PI on HIF1α protein expression levels in the MP (**a**) and FP (**b**) at e15 and e20, detected by Western blotting. (**a**) HIF1α protein levels: e20: ** *p* < 0.01 vs. control (Student’s test).

Using Western blotting, we observed no changes in HIF1α protein levels in the MP (Figure 1a) and FP (Figure 1b) of the MH group compared with controls at both e15 and e20. Similarly, PI did not affect HIF1α protein levels in the MP (Figure 1a) or FP (Figure 1b) at e15. However, at e20, a significant decrease in HIF1α protein expression was observed in the MP (but not FP) in the PI group (Figure 1a, **—*p* = 0.007 Student’s test).

**Figure 2:**
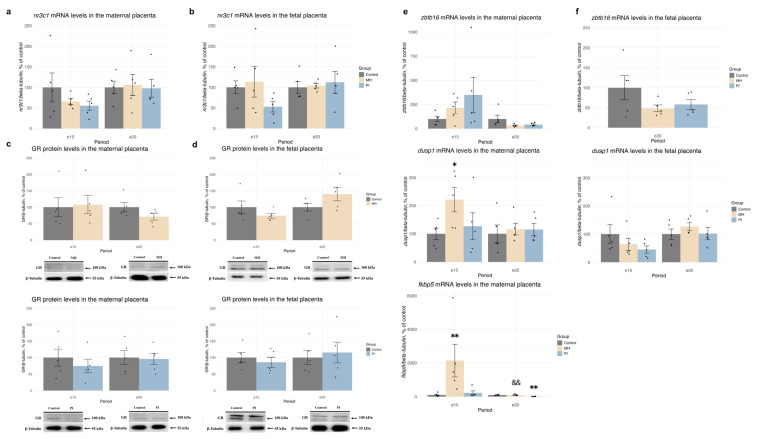
The effect of MH and PI on mRNA levels of the glucocorticoid receptor (*nr3c1*) in the MP (**a**) and FP (**b**) at e15 and e20, detected by RT PCR. The effect of MH and PI on GR protein expression levels in the MP (**c**) and FP (**d**) at e15 and e20, detected by Western blotting. The effect of MH and PI on mRNA levels of the glucocorticoid-dependent genes *ztb16*, *dusp1*, and *fkbp5* in the MP (**e**) and FP (**f**) at e15 and e20, detected by RT PCR. (**e**) mRNA levels of *dusp1*: e15: * *p* < 0.05 vs. control (Student’s test). mRNA levels of *fkbp5*: e15: ** *p* < 0.01 vs. control (Kruskal-Wallis test, Dunn’s test). e20 ** *p* < 0.01 vs. control (one-way ANOVA, Tukey’s test), && *p* < 0.01 between MH and PI (one-way ANOVA, Tukey’s test).

Neither MH nor PI affected the relative mRNA levels of np3c1 or GR protein levels in either MP (Figure 2a,c) or FP (Figure 2b,d) at e15 and e20. To assess the transcriptional activity of glucocorticoid receptors, we analyzed the relative mRNA levels of three genes: *zbtb16*, *dusp1*, and *fkbp5* (Figure 2e,f). In the MP, MH did not affect the expression of *zbtb16* at e15 and e20, but it significantly increased the expression of *dusp1* (Figure 2e, MH vs. control, *p* = 0.03, Student’s test) and *fkbp5* (Figure 2e, Kruskal-Wallis *p* = 0.008, MH vs. control *p* = 0.007, Dunn’s test) at e15, with no effect at e20. In the PI group, there was no effect on the expression of *zbtb16* or *dusp1* at either e15 or e20. However, *fkbp5* expression was decreased at e20 (Figure 2e, one-way ANOVA F (2, 12) = 14.85, *p* = 0.0005, MH vs. control *p* = 0.0015; MH vs. PI *p* = 0.001 Tukey’s test). When studying GR-dependent transcription in the FP, only the gene *dusp1* was detected at e15 and e20, and *zbtb16* at e20 (Figure 2f). Neither MH nor PI affected the expression of these genes at the documented time points.

**Figure 3:**
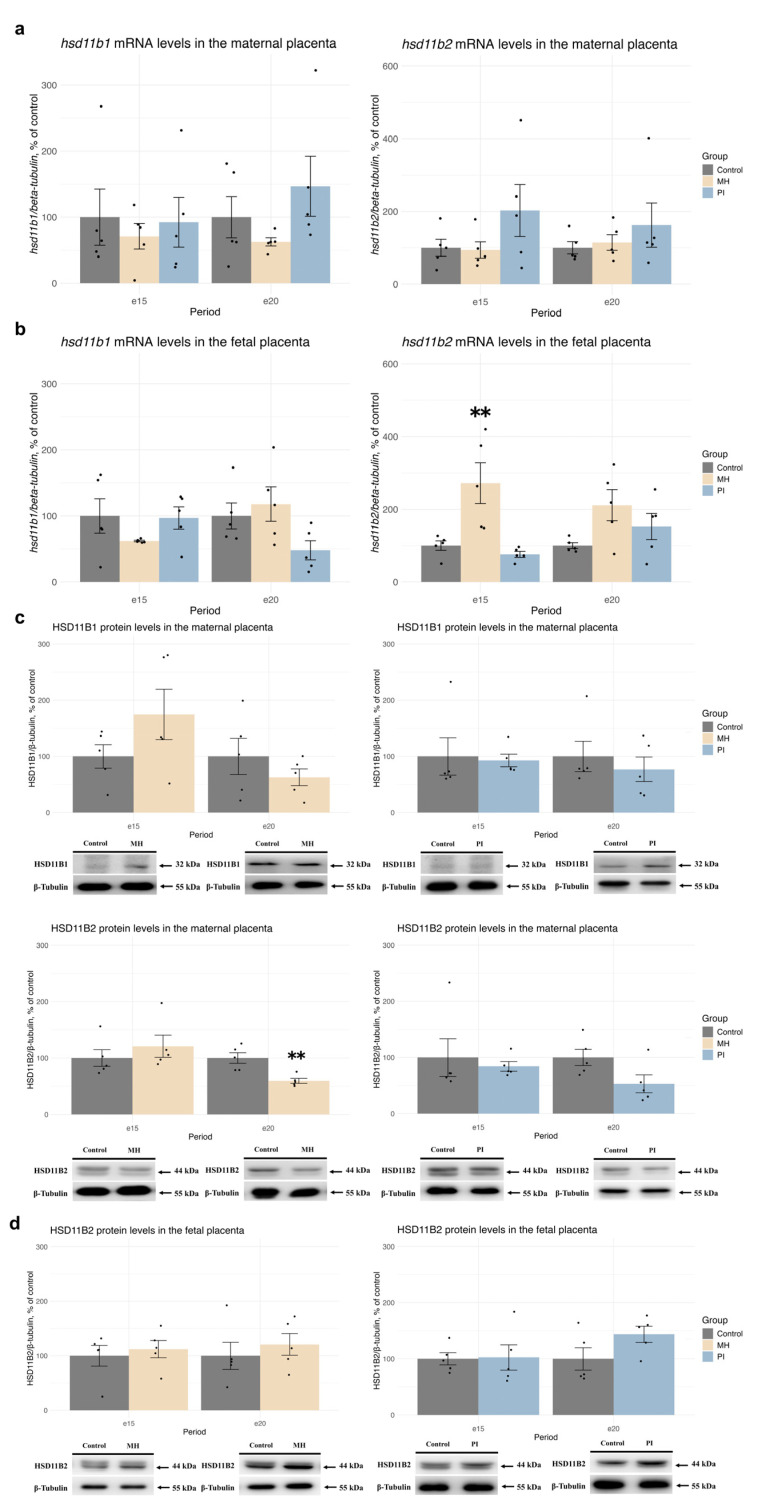
The effect of MH and PI on mRNA levels of the *hsd11b1* and *hsd11b2* in the MP (**a**) and FP (**b**) at e15 and e20, detected by RT PCR. The effect of MH and PI on the protein expression levels of HSD11B1 and HSD11B2 in the MP (**c**) and HSD11B2 in the FP (**d**) at e15 and e20, detected by Western blotting. (**b**) mRNA levels of *hsd11b2*: e15: ** *p* < 0.01 vs. control (Welch ANOVA, Dunnett’s test). (**c**) HSD11B2 protein levels: e20: ** *p* < 0.01 vs. control (Student’s test).

We also examined the relative mRNA and protein levels of HSD11B1, the enzyme responsible for synthesizing corticosterone from 11-dehydrocorticosterone in rats, and HSD11B2, which oxidizes corticosterone to 11-dehydrocorticosterone, in the MP and FP at e15 and e20 (Figure 3). In the MP, neither MH nor PI affected the mRNA expression levels of *hsd11b1* or *hsd11b2* (Figure 3a), and no changes in HSD11B1 protein levels were observed at any time point (Figure 3c). HSD11B2 protein levels in the MP were also unchanged in response to MH or PI at e15 (Figure 3c). However, at e20 the MH (but not PI) group showed a significant decrease in HSD11B2 protein expression (Figure 3c, MH vs. control, *p* = 0.004, Student’s test).

In the FP, neither MH nor PI affected the mRNA expression levels of *hsd11b1* at e15 and e20 (Figure 3b). However, MH (but not PI) caused an increase in *hsd11b2* mRNA expression levels at e15 (Figure 3b, Welch ANOVA F (2, 6.71) = 6.2735, *p* = 0.02909, MH vs. control *p* = 0.006, Dunnet’s test). At e20, no changes in *hsd11b2* mRNA expression were observed in either the MH or PI groups (Figure 3b). The HSD11B1 protein was undetectable in the FP, and no changes in HSD11B2 protein expression were found in response to MH or PI (Figure 3d).

### 2.2. Activity of the Glucocorticoid System in Brain Development During MH or PI

**Figure 4:**
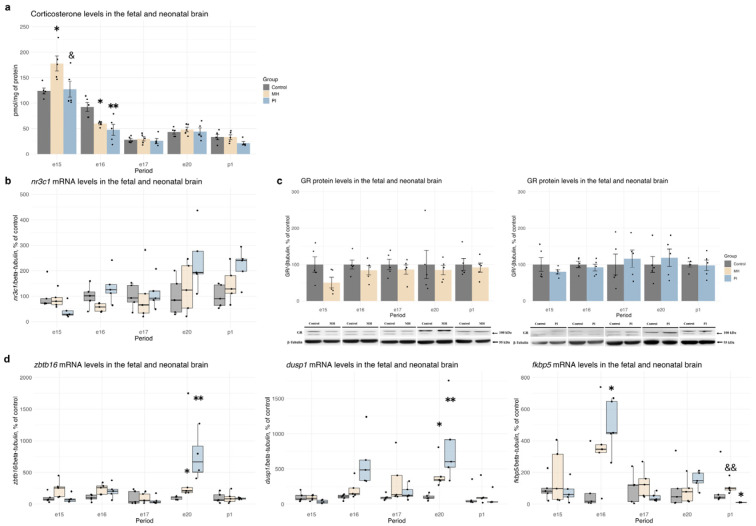
The effect of MH and PI on the corticosterone levels (**a**) in the brain at e15, e16, e17, e20 and p1, detected by ELISA. The effect of MH and PI on mRNA levels of the glucocorticoid receptor (*nr3c1*) (**b**) in the brain at e15, e16, e17, e20 and p1, detected by RT PCR. The effect of MH and PI on GR protein expression levels (**c**) in the brain at e15, e16, e17, e20 and p1, detected by Western blotting. The effect of MH and PI on mRNA levels of the glucocorticoid-dependent genes *ztb16*, *dusp1*, and *fkbp5* (**d**) in the brain at e15, e16, e17, e20 and p1, detected by RT PCR. (**a**) Corticosterone levels: e15, * *p* < 0.05 vs. control (one-way ANOVA, Tukey’s test); & *p* < 0.05 between MH and PI (one-way ANOVA, Tukey’s test). e16, * *p* < 0.05 vs. control (one-way ANOVA, Tukey’s test); ** *p* < 0.01 vs. control (one-way ANOVA, Tukey’s test). (**d**) mRNA levels of *zbtb16*: e20, * *p* < 0.05 vs. control (Kruskal-Wallis, Dunn’s test); ** *p* < 0.01 vs. control (Kruskal-Wallis, Dunn’s test). mRNA levels of *dusp1*: e20, * *p* < 0.05 vs. control (Kruskal-Wallis, Dunn’s test); ** *p* < 0.01 vs. control (Kruskal-Wallis, Dunn’s test). mRNA levels of *fkbp5*: e16, * *p* < 0.05 vs. control (Kruskal-Wallis, Dunn’s test); p1, * *p* < 0.05 vs. control (Kruskal-Wallis, Dunn’s test); && *p* < 0.01 between MH and PI (Kruskal-Wallis, Dunn’s test).

When examining corticosterone levels in embryonic brains, we observed a significant increase in corticosterone concentration 1 day after MH at e15 (Figure 4a, one-way ANOVA F (2, 12) = 13.33, *p* = 0.019, MH vs. control *p* = 0.02; PI vs. MH *p* = 0.03). Additionally, at e16, a significant decrease in corticosterone concentration was detected in the brains of both MH and PI embryos (Figure 4a, one-way ANOVA F (2, 12) = 8.124, *p* = 0.005, MH vs. control *p* = 0.02; PI vs. control *p* = 0.005). No significant differences in corticosterone levels were found at e17, e20, or p1 between the MH, PI, and control groups. Regarding the relative mRNA expression of *nr3c1* in the brain, we did not observe significant changes in the MH or PI groups compared to controls at e15, e16, e17, e20, and p1 (Figure 4b). Similarly, no significant alterations were detected in GR protein expression (Figure 4c). To assess the transcriptional activity of glucocorticoid receptors, we analyzed the relative mRNA levels of three genes: *zbtb16*, *dusp1,* and *fkbp5* (Figure 4d). We found a significant increase in *zbtb16* (Figure 4d, Kruskal-Wallis *p* = 0.004, MH vs. control *p* = 0.05; PI vs. control *p* = 0.003, Dunn’s test) and *dusp1* (Figure 4d, Kruskal-Wallis *p* = 0.005, MH vs. control *p* = 0.05; PI vs. control *p* = 0.004, Dunn’s test) mRNA expression in both the MH and PI groups at e20. Additionally, *fkbp5* expression increased in the PI group at e16 (Figure 4d, Kruskal-Wallis *p* = 0.04, PI vs. control *p* = 0.039, Dunn’s test), but decreased at p1 (Figure 4d, Kruskal-Wallis *p* = 0.005, PI vs. control *p* = 0.05; PI vs. MH *p* = 0.004 Dunn’s test).

**Figure 5:**
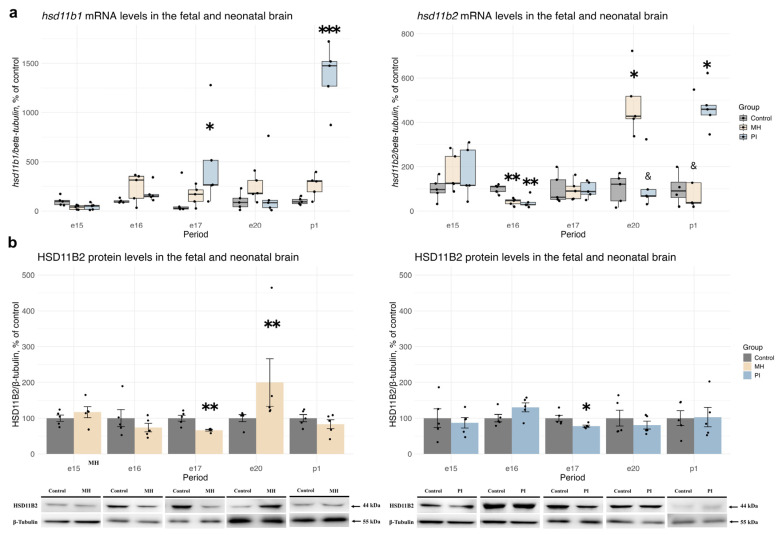
The effect of MH and PI on mRNA levels of *hsd11b1* and *hsd11b2* (**a**) in the brain at e15, e16, e17, e20 and p1, detected by RT PCR. The effect of MH and PI on the protein expression levels of the HSD11B2 (**b**) in the brain at e15, e16, e17, e20 and p1, detected by Western blotting. (**a**) mRNA levels of *hsd11b1*: e17, * *p* < 0.05 vs. control (Kruskal-Wallis, Dunn’s test); p1, *** *p* < 0.001 vs. control (Kruskal-Wallis, Dunn’s test). mRNA levels of *hsd11b2*: e16, ** *p* < 0.01 vs. control (one-way ANOVA, Tukey’s test); e20, * *p* < 0.05 vs. control (Kruskal-Wallis, Dunn’s test); & *p* < 0.05 between MH and PI (Kruskal-Wallis, Dunn’s test); p1, * *p* < 0.05 vs. control (Welch ANOVA, Dunnett’s test); & *p* < 0.05 between MH and PI (Welch ANOVA, Dunnett’s test). (**b**) HSD11B2 protein levels: e17, * *p* < 0.05 vs. control (Student’s test), ** *p* < 0.01 vs. corresponding control (Student’s test); e20, ** *p* < 0.01 vs. control (Mann-Whitney’s test).

We also analyzed the relative mRNA and protein levels of HSD11B1 and HSD11B2, enzymes involved in local corticosterone synthesis and oxidation, in rat brain during embryonic (e15, e16, e17, e20) and early postnatal development (p1) (Figure 5). MH did not affect *hsd11b1* mRNA expression at any of the studied time points, whereas PI caused an increase in *hsd11b1* mRNA at e17 (Figure 5a, Kruskal-Wallis *p* = 0.05, PI vs. control *p* = 0.02) and p1 (Figure 5a, Kruskal-Wallis *p* = 0.003, PI vs. control *p* = 0.0006) compared to controls. HSD11B1 protein expression in embryonic and newborn rat brain was undetectable. Both MH and PI resulted in a decrease in *hsd11b2* mRNA expression at e16 (Figure 5a, one-way ANOVA F (2, 12) = 13.33, *p* = 0.0008, MH vs. control *p* = 0.002; PI vs. control *p* = 0.001 Tukey’s test). Additionally, MH at e20 (Figure 5a, Kruskal-Wallis *p* = 0.009, MH vs. control *p* = 0.02; MH vs. PI *p* = 0.02, Dunn’s test) and PI at p1 (Figure 5a, Welch ANOVA F (2, 7.039) = 18.405, *p* = 0.001, PI vs. control *p* = 0.008, Dunnet’s test) showed an increase in *hsd11b2* mRNA expression. The decrease in *hsd11b2* transcription in response to MH and PI at e16 was accompanied by a decrease in HSD11B2 protein expression at e17 (Figure 5b, MH vs. control *p* = 0.004; PI vs. control *p* = 0.03, Student’s test). By e20, HSD11B2 protein expression had increased in the MH group compared to the control group (Figure 5b, *p* = 0.007, Mann-Whitney’s test).

### 2.3. Expression of HIF1α, HIF1-Dependent Transcription, and Anaerobic Glycolysis Activity in Brain Development During MH or PI

**Figure 6:**
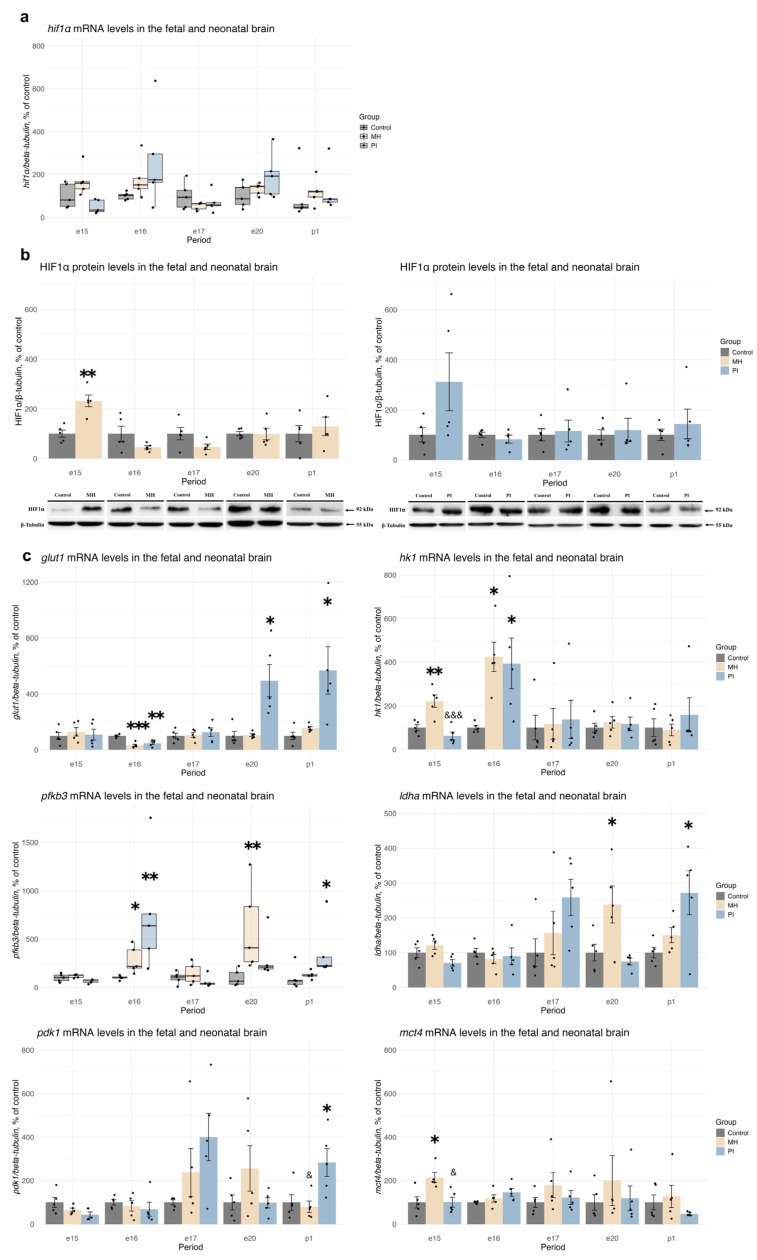
The effect of MH and PI on mRNA levels of *hif1α* (**a**) in the brain at e15, e16, e17, e20 and p1, detected by RT PCR. The effect of MH and PI on HIF1α protein expression levels (**b**) in the brain at e15, e16, e17, e20 and p1, detected by Western blotting. The effect of MH and PI on mRNA levels of the HIF1-dependent genes *glut1*, *hk1*, *pfkb3*, *ldha*, *pdk1*, and *mct4* (**c**) in the brain at e15, e16, e17, e20 and p1, detected by RT PCR. (**b**) HIF1α protein levels: e15: ** *p* < 0.01 vs. control (Student’s test). (**c**) mRNA levels of *glut1*: e16, ** *p* < 0.01 vs. control (one-way ANOVA, Tukey’s test), *** *p* < 0.001 vs. control (one-way ANOVA, Tukey’s test); e20, * *p* < 0.05 vs. control (Welch ANOVA, Dunnett’s test); p1, * *p* < 0.05 PI vs. control (Welch ANOVA, Dunnett’s test). mRNA levels of *hk1*: e15, ** *p* < 0.01 vs. control (one-way ANOVA, Tukey’s test); &&& *p* < 0.001 between MH and PI (one-way ANOVA, Tukey’s test); e16, * *p* < 0.05 vs. control (Welch ANOVA, Dunnett’s test). mRNA levels of *pfkb3*: e16, * *p* < 0.05 vs. control (Kruskal-Wallis, Dunn’s test), ** *p* < 0.01 vs. control (Kruskal-Wallis, Dunn’s test); e20, ** *p* < 0.01 vs. control (Kruskal-Wallis, Dunn’s test); p1, * *p* < 0.05 vs. control (Kruskal-Wallis, Dunn’s test). mRNA levels of *ldha*: e20, * *p* < 0.05 vs. control (one-way ANOVA, Tukey’s test); p1, * *p* < 0.05 vs. control (Welch ANOVA, Dunnett’s test). mRNA levels of *pdk1*: p1, * *p* < 0.05 vs. control (one-way ANOVA, Tukey’s test); & *p* < 0.05 between MH and PI (one-way ANOVA, Tukey’s test). mRNA levels of *mct4*: e15, * *p* < 0.05 vs. control (one-way ANOVA, Tukey’s test); & *p* < 0.05 between MH and PI (one-way ANOVA, Tukey’s test).

In the embryonic brains of the MH group (but not the PI group), we observed an increase in HIF1α protein levels at e15 compared with control (Figure 6b, *p* = 0.002, Student’s test), despite the lack of changes in *hif1α* mRNA expression levels (Figure 6a). During subsequent embryonic (e16, e17, e20) and early postnatal (p1) development, neither MH nor PI induced changes in HIF1α mRNA or protein expression compared with controls.

To study HIF1-dependent transcription, we analyzed the relative mRNA expression of *glut1*, *hk1*, *pfkb3*, *ldha*, *pdk1*, *mct4* in embryonic and newborn brains (Figure 6c). We found a decrease in *glut1* mRNA expression in both groups at e16 (Figure 6c, one-way ANOVA F (2, 12) = 19.1, *p* = 0.0001, MH vs. control *p* = 0.0002; PI vs. control *p* = 0.001, Dunnet’s test). However, an increase in *glut1* mRNA expression was observed in the PI group at e20 (Figure 6c, Welch ANOVA F (2, 5.83) = 5.1576, *p* = 0.05, PI vs. control *p* = 0.05, Dunnet’s test) and p1 (Figure 6c, Welch ANOVA F (2, 6.18) = 4.3926, *p* = 0.05, PI vs. control *p* = 0.01, Dunnet’s test).

We also noted an increase in *hk1* mRNA expression in the MH group at e15 (Figure 6c, Welch ANOVA F (2, 5.48) = 12.649, *p* = 0.008, MH vs. control *p* = 0.02; PI vs. control *p* = 0.03, Dunnet’s test) and e16 (Figure 6c, one-way ANOVA F (2, 12) = 15.81, *p* = 0.0004, MH vs. control *p* = 0.003, PSH vs. PI *p* = 0.0004, Tukey’s test). Additionally, *pfkb3* mRNA expression increased in the MH group at e16 (Figure 6c, Kruskal-Wallis *p* = 0.005, MH vs. control *p* = 0.05, Dunn’s test) and e20 (Figure 6c, Kruskal-Wallis *p* = 0.009, MH vs. control *p* = 0.007, Dunn’s test), as well as in the PI group at e16 (Figure 6c, PI vs. control *p* = 0.004, Dunn’s test) and at p1 (Figure 6c, Kruskal-Wallis *p* = 0.018, PI vs. control *p* = 0.017, Dunn’s test).

An increase in *pdk1* mRNA expression was observed in the PI group at p1 (Figure 6c), one-way ANOVA F (2, 12) = 6.201, *p* = 0.014, PI vs. control *p* = 0.03, PSH vs. PI *p* = 0.02, Tukey’s test), and an increase in *mct4* mRNA expression was found in the MH group at e15 (Figure 6c, one-way ANOVA F (2, 12) = 7.475, *p* = 0.007, MH vs. control *p* = 0.01; PSH vs. PI *p* = 0.01, Tukey’s test). Moreover, *ldha* mRNA expression increased in the MH group at e20 (Figure 6c, one-way ANOVA F (2, 12) = 6.597, *p* = 0.011, MH vs. control *p* = 0.03, Tukey’s test) and in the PI group at p1 (Figure 6c, Welch ANOVA F (2, 7.043) = 4.3203, *p* = 0.05, PI vs. control *p* = 0.01, Dunnet’s test).

**Figure 7:**
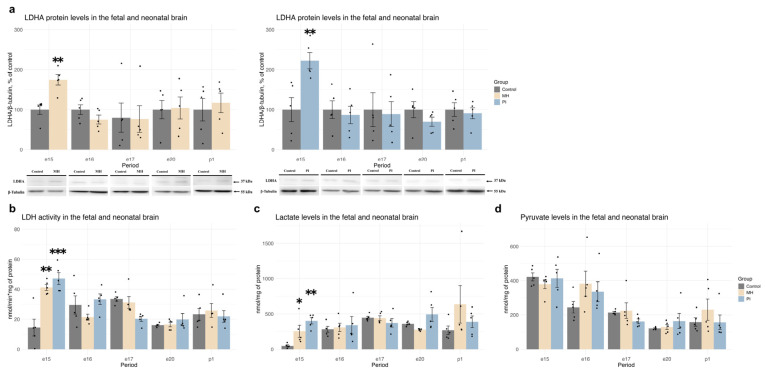
The effect of MH and PI on LDHA protein expression levels (**a**) in the brain at e15, e16, e17, e20 and p1, detected by Western blotting. The effect of MH and PI on LDH activity (**b**), lactate (**c**) and pyruvate (**d**) levels in the brain at e15, e16, e17, e20 and p1, detected by colorimetric tests. (**a**) LDHA protein levels: e15, ** *p* < 0.01 MH vs. control (Student’s test), ** *p* < 0.01 PI vs. control (Student’s test). (**b**) LDH activity: e15, ** *p* < 0.01 vs. control (one-way ANOVA, Tukey’s test), *** *p* < 0.001 vs. control (one-way ANOVA, Tukey’s test). (**c**) Lactate levels: e15, * *p* < 0.05 vs. control (one-way ANOVA, Tukey’s test), ** *p* < 0.01 vs. control (one-way ANOVA, Tukey’s test).

In both groups, an increase in LDHA protein expression was also observed at e15 (Figure 7a, MH vs. control, *p* = 0,002; PI vs. control *p =* 0.009, Student’s test), accompanied by elevated LDH enzymatic activity (Figure 7b, one-way ANOVA F (2, 12) = 17.96, *p* = 0.0002, MH vs. control *p* = 0.001; PI vs. control *p* = 0.0003, Tukey’s test) and increased lactate concentration (Figure 7c, one-way ANOVA F (2, 12) = 10,01, *p* = 0.002, MH vs. control *p* = 0.05; PI vs. control *p* = 0.002, Tukey’s test), without affecting pyruvate concentration (Figure 7d). During further development (e16, e17, e20, p1), no significant effects of MH and PI were detected on LDHA protein expression, LDH enzymatic activity, or lactate and pyruvate concentrations.

### 2.4. Activity of the Pentose Phosphate Pathway and Redox State in Brain Development During MH or PI

**Figure 8:**
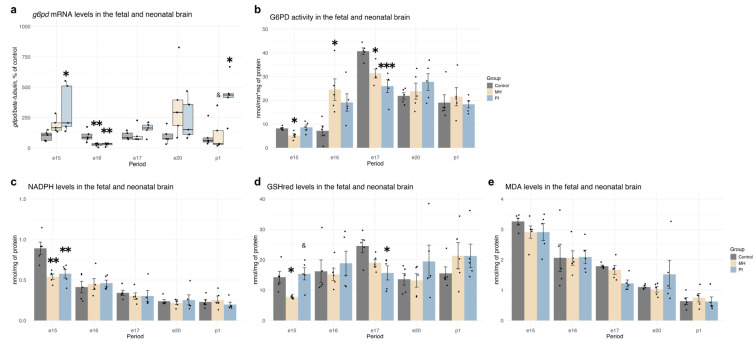
The effect of MH and PI on mRNA levels of *g6pd* (**a**) in the brain at e15, e16, e17, e20 and p1, detected by RT PCR. The effect of MH and PI on G6PD activity (**b**), NADPH (**c**), GSHred (**d**) and MDA (**e**) levels in the brain at e15, e16, e17, e20 and p1, detected by colorimetric tests. (**a**) mRNA levels of *g6pd*: e15, * *p* < 0.05 vs. control (Welch ANOVA, Dunnett’s test); e16, ** *p* < 0.01 vs. control (one-way ANOVA, Tukey’s test); p1, * *p* < 0.05 vs. control (Kruskal-Wallis, Dunn’s test); & *p* < 0.05 between MH and PI (Kruskal-Wallis, Dunn’s test). (**b**) G6PD activity: e15, * *p* < 0.05 vs. control (one-way ANOVA, Tukey’s test); e16, * *p* < 0.05 vs. control (Welch ANOVA, Dunnett’s test); e17, * *p* < 0.05 vs. control (one-way ANOVA, Tukey’s test), *** *p* < 0.001 vs. control (one-way ANOVA, Tukey’s test). (**c**) NADPH levels: e15, ** *p* < 0.01 vs. control (one-way ANOVA, Tukey’s test). (**d**) GSHred levels: e15: * *p* < 0.05 vs. control (one-way ANOVA, Tukey’s test); & *p* < 0.05 between MH and PI (one-way ANOVA, Tukey’s test). e17: * *p* < 0.05 vs. control (one-way ANOVA, Tukey’s test).

In our analysis of pentose phosphate pathway activity in the developing brain, we observed an increase in *g6pd* mRNA expression in both the MH and PI groups at e15 (Figure 8a, Kruskal-Wallis *p* = 0.046, MH vs. control *p* = 0.047; PI vs. control *p* = 0.007, Dunn’s test), followed by a decrease at e16 (Figure 8a, one-way ANOVA F (2, 12) = 9.412, *p* = 0.003, MH vs. control *p* = 0.005; PI vs. control *p* = 0.008, Tukey’s test). Additionally, an increase was observed in the PI group at p1 (Figure 8a, Kruskal-Wallis *p* = 0.016, PI vs. control *p* = 0.05; PI vs. PSH *p* = 0.02, Dunn’s test). No significant changes in *g6pd* mRNA expression were detected in either group during later developmental stages (e17, e20, p1).

Functional analysis revealed a decrease in G6PD enzymatic activity in the MH group (but not in the PI group) at e15 compared to the control group (Figure 8b, one-way ANOVA F (2, 12) = 6.497, *p* = 0.01, MH vs. control *p* = 0.03; PI vs. MH *p* = 0.01, Tukey’s test). By e16, G6PD enzymatic activity increased in the MH group compared to the control group (Figure 8b, Welch ANOVA F (2, 7.1574) = 7.7279, *p* = 0.016, MH vs. control *p* = 0.009; PI vs. control *p* = 0.008, Dunnet’s test). At e17, G6PD enzymatic activity in the control group significantly increased over time (two-way ANOVA Group * Period F (8, 60) = 5.114, *p* < 0.0001, control e17 vs. control e16 *p* < 0.0001) and was higher than in the MH (Figure 8b, one-way ANOVA F (2, 12) = 13.02, *p* = 0.009, MH vs. control *p* = 0.01) and PI groups (Figure 8b, PI vs. control *p* = 0.0007). No significant differences in G6PD activity were found at e20 or p1 in either group compared to controls.

Both MH and PI groups exhibited a decrease in NADPH concentration, a product of the pentose phosphate pathway, at e15 (Figure 8c, one-way ANOVA F (2, 12) = 11.24, *p* = 0.001, MH vs. control *p* = 0.003; PI vs. control *p* = 0.005, Tukey’s test) with levels returning to normal at e16, and no further changes observed at e17, e20, or p1.

The reduction in G6PD enzymatic activity and NADPH concentration in MH embryos at e15 was accompanied by a decrease in GSHred levels (Figure 8d, one-way ANOVA F (2, 12) = 6.728, *p* = 0.011, MH vs. control *p* = 0.03; PI vs. MH *p* = 0.01). However, by e17, only the PI group showed a notable decrease in GSHred compared to the control group, with no similar reduction observed in the MH group (Figure 8d, one-way ANOVA F (2, 12) = 5.217, *p* = 0.023, PI vs. control *p* = 0.02). No significant changes in GSHred concentration were detected during later stages (e16, e20, and p1) either in the MH or in the PI group. Despite these changes in pentose phosphate pathway activity and redox status, neither MH nor PI affected the concentration of MDA, a marker of lipid peroxidation (Figure 8e).

## 3. Discussion

Environmental factors play a critical role in shaping the development of organisms [2,3,7,26,27,28]. The conditions experienced by an embryo or fetus influence epigenetic programming [10,20], providing key information about the potential future environment. A normal course of pregnancy ensures an efficient supply of energy substrates essential for intensive processes such as cell proliferation, migration, and neuronal network formation [29]. While a limitation in steroid hormones influx is considered normal until the late stages of pregnancy, during late pregnancy, the regulated influx of steroid hormones, particularly glucocorticoids, becomes crucial for terminal differentiation of neuronal cells and lung maturation [30,31]. Disruption in oxygen supply to the embryo can significantly impair energy metabolism, leading to growth retardation [32,33,34,35]. Excessive glucocorticoid exposure can also alter tissue-specific gene expression profiles, with potential lifelong consequences [22,36].

In both hypoxic models employed in this study, we observed a rapid increase in the transcriptional activity of HIF1α (the key regulator of hypoxic adaptation) in the embryonic brain, without notable changes in the maternal and fetal placental compartments. This was accompanied by elevated activity of lactate dehydrogenase A (the enzyme responsible for anaerobic glycolysis under oxygen-deficient conditions) and increased lactate levels, which is the product of lactate dehydrogenase A activity [37,38]. Although enhanced anaerobic glycolysis is a crucial protective mechanism, direct stimulation of this pathway, alongside HIF1α-dependent suppression, could have delayed adverse effects on the cellular antioxidant defense system by inhibiting the pentose phosphate pathway of glucose metabolism [38,39,40]. Placental ischemia was found to diminish the efficiency of NADPH production, likely through increased glycolytic activity without impacting the activity of glucose-6-phosphate dehydrogenase or cellular redox status at e15. Conversely, maternal hypoxia led to a reduction in G6PD activity and NADPH levels, along with impaired downstream redox processes, as evidenced by decreased concentrations of reduced glutathione, while not affecting the heightened production of reactive oxygen species during the embryonic period. Our previous studies have shown that elevated HIF1α levels induced by maternal hypoxia persist throughout the lifespan of the offspring, contributing to hippocampal oxidative stress [38]. This condition is associated with dysfunction of the pentose phosphate pathway and increased activation of anaerobic glycolysis, which are factors implicated in the development of depressive-like behavior in adulthood and the acceleration of aging [38,41,42].

The complex interplay between HIF1 activity and the nonspecific activation of the glucocorticoid stress response is of particular interest [43,44,45]. Glucocorticoids, acting through their receptors, can enhance HIF1-dependent transcription while simultaneously exerting a delayed inhibitory effect on HIF1 at the transcriptional level [46]. The interplay between HIF and GR during brain development can increase oxidative stress in maturing neurons and cause delayed disturbances in vascularization, as demonstrated in numerous studies [34,47]. The redox imbalance may compromise the cellular environment necessary for proper neurogenesis and synaptic refinement [48]. Moreover, excessive glucocorticoid levels have been implicated in altered neuronal differentiation and connectivity. Thus, the combined effects of hypoxia-induced metabolic reprogramming and glucocorticoid exposure could create a suboptimal niche for developing neurons, ultimately influencing the establishment of neural circuits and predisposing offspring to neurological and psychiatric conditions later in life [49,50]. A key distinction between the models investigated here lies in the presence of a maternal glucocorticoid stress response, which is accompanied by an excessive influx of corticosterone through the placenta. This response, observed in the model of maternal hypoxia but not in prenatal ischemia, leads to increased glucocorticoid-dependent transcription in the maternal placenta and the developing brain at e15. By e16, corticosterone levels in the embryonic brain had decreased in both models, likely due to different mechanisms. In the case of maternal hypoxia, this decrease may be attributed to the negative feedback loop of glucocorticoids, while in placental ischemia, the ischemic condition itself likely reduced corticosterone availability. At e15, hippocampal formation is underway in the fetal brain [7]. Despite this, we did not observe alterations in glucocorticoid receptor protein expression or significant changes in its transcription in the brain, possibly due to averaging across the entire brain. It is plausible that premature exposure to glucocorticoid receptors during early hippocampal maturation sets the stage for lifelong disruptions in their transcription and translation within the hippocampus. These disruptions, along with altered circadian dynamics of serum glucocorticoid levels, have been linked to depressive-like phenotypes, as described by previous works in cases where fetal hypoxia is combined with maternal stress [2,41,42,51,52], as well as in mono-models of maternal stress or their hormonal imitations [53,54,55]. Conversely, while placental ischemia induces significant alterations in HIF1-dependent metabolism throughout the embryonic period and delays physical development [56], the absence of glucocorticoid involvement in this process appears to mitigate severe psychoneurological consequences in postnatal ontogeny, primarily affecting the function of visceral organs [21,57,58]. Notably, maternal hypoxia and placental ischemia had distinct effects on the postnatal expression of glucocorticoid receptors and glucocorticoid-dependent transcription in the developing brain [17,22,51]. These effects, along with alterations in glucocorticoid-related gene expression during embryogenesis, were influenced by complex and difficult-to-differentiate responses to both conditions, even as glucocorticoid innervation remained largely unaltered compared to controls. Despite these significant changes in the brain, no significant changes in the expression of glucocorticoid receptors were observed in either the maternal or fetal parts of the placenta following maternal hypoxia or placental ischemia. The manifestation of the glucocorticoid-dependent transcriptional program may be influenced by fluctuations in corticosterone concentration in maternal blood or by challenges in its availability during placental ischemia.

Alterations in the balance between anaerobic glycolysis and the pentose phosphate pathway, along with glucocorticoid involvement in the HIF1-dependent transcriptional program, can significantly impact local corticosteroid synthesis and oxidation processes [46,59]. Specifically, HSD11B1 facilitates the synthesis of cortisol in humans and corticosterone in rodents by utilizing NADPH [59,60], a product of the pentose phosphate pathway [61]. Conversely, HSD11B2 oxidizes cortisol/corticosterone using NAD, a crucial substrate for both glycolysis and mitochondrial energy metabolism [59,62,63]. While no substantial changes in the expression of these enzymes were detected in either the maternal or fetal placental tissues and the alterations observed in the embryonic brain appear to be adaptive responses to corticosterone fluctuations, the robust activation of glycolysis and suppression of the pentose phosphate pathway in the postnatal brain may lead to significant disruptions in the plasticity of local glucocorticoid signaling, which is regulated by these enzymes [38,59].

A detailed examination of HIF1α expression and the metabolic processes in the developing brain mediated by this protein, alongside the activity of glucocorticoid penetration, corticosterone receptor expression, glucocorticoid-dependent transcription, and the dynamics of enzymes involved in local glucocorticoid synthesis and degradation under the influence of maternal hypoxia and placental ischemia, reveals both general oxygen-dependent effects on the developing brain and significant differences in the stability of observed changes. These differences appear to depend on the presence or absence of a maternal stress component. The findings of this study contribute to a deeper understanding of brain development and offer insights into the differential impacts of hypoxic and stress-related environmental factors on the formation of predispositions to specific neurological outcomes. Furthermore, combining a maternal stress model of hypoxia with fetal hypoxia may serve as an effective approach for elucidating cause-and-effect relationships.

While this study offers new insights into the interplay between hypoxia-induced metabolic reprogramming and glucocorticoid signaling during brain development, several limitations warrant mention. First, comparing the intermittent MH model with the chronic PI model does not fully account for potential differences in compensatory vascular responses and reoxygenation effects. Refining experimental techniques to more precisely monitor and control fetal oxygenation would help delineate the dose-dependent effects of hypoxia on metabolic and endocrine pathways. Second, our current findings are based on a single rodent model; exploring additional species or strains known to differ in their susceptibility to prenatal stress could enhance the translational potential of our results. Finally, while we have emphasized global responses, investigating sex-specific effects, as well as the long-term behavioral and cognitive outcomes in adulthood, will be essential for understanding the full impact of fetal hypoxia and maternal stress on the neurological health of the progeny.

## 4. Materials and Methods

### 4.1. Animals

The study was carried out using animals from the CCU “Biocollection of laboratory mammals of different taxonomic affiliation” of the Pavlov Institute of Physiology of RAS. Adult pregnant female Wistar rats, aged 12–13 weeks and weighing 220–250 g, along with their embryonic (e15, e16, e17, e20) and newborn (p1) progeny without sex definition were utilized. All experimental procedures were performed in compliance with The Guidelines for Reporting Animal Research [64] and were approved by the Ethical Committee for the Use of Animal Subjects at the Pavlov Institute of Physiology (protocol no. 08/02 of 2 August 2022).

### 4.2. Maternal Hypoxia

**Figure 9:**
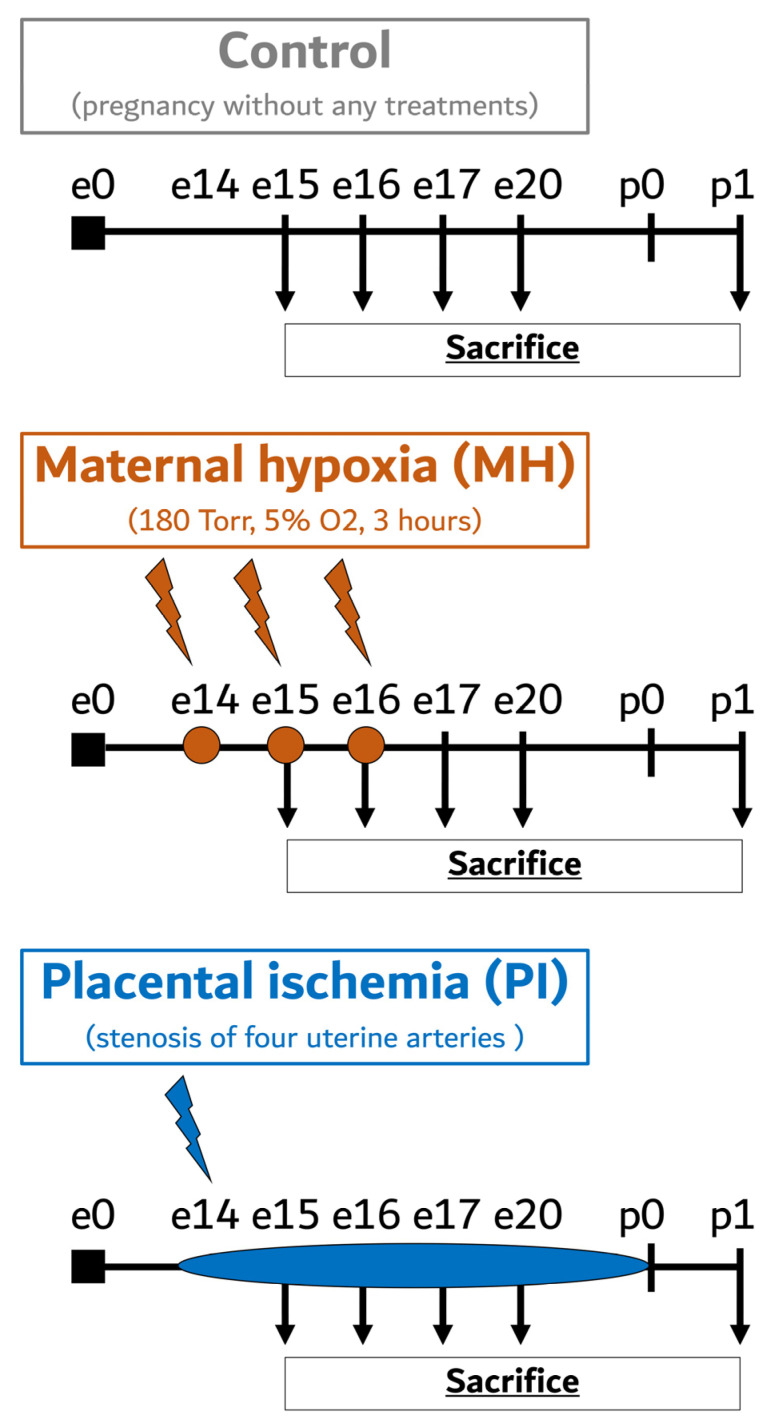
Schematic outline of the experimental study design. MH, maternal hypoxia; PI, placental ischemia; e0–e20, embryonic days; p0, day of birth; p1, first postnatal day.

The maternal hypoxia (MH) model reported in our previous studies was used as a reliable model combining the fetal hypoxia and maternal stress response during pregnancy (Figure 9) [11,22,23].

To model MH, we used a flow-type hypobaric chamber at a temperature of 20 to 25 °C in which atmospheric pressure was gradually reduced to 180 Torr reaching 5% of oxygen content (equivalent to 11,000 m above sea level) during 20 min. The experimental chamber was partitioned into six distinct compartments, thereby facilitating the simultaneous modeling of MH on six pregnant rats. After 3 h of treatment, the oxygen content was returned to normal within 20 min. Pregnant dams were treated under such conditions for three consecutive days (e14–e16) with an interval of 24 h between sessions. The mortality rate in the hypobaric chamber was around 15%.

### 4.3. Placental Ischemia

To compare the fetal hypoxia effects with the consequences of fetal hypoxia combined with maternal stress, we utilized an experimental group exposed to placental ischemia (PI) from the 14th day of pregnancy (e14), matching it with the MH group in all experiments (Figure 9). PI was induced via arterial stenosis according to Tsuji’s protocol [21], with some modifications [51]. Pregnant dams were anesthetized using an Animal Anesthesia Machine (RWD Live Science, San-Diego, CA, USA) with isoflurane. The anesthetic gas was delivered at a flow rate of 0.5 L/min, inducing narcosis with an initial concentration of 4% and maintaining it at 2% throughout the procedure. Four feeding arteries to the uterus, that is, the bilateral uterine and ovarian arteries, were exposed and separated from the veins running along each artery to provide space for microcoil insertion. Under a microscope, each artery was gently lifted with a silk suture, and a microcoil (φ = 0.21 mm) made of surgical steel (Technopark, Saint-Petersburg, Russia) was wrapped around the artery by rotating five times under a microscope. Blood flow restriction inside the artery was visually confirmed based on the whitening of the artery.

### 4.4. Sample Preparation

To collect the samples for further analysis, tissues from the embryonic (e15, e16, e17, and e20) and newborn (p1) brains and embryonic (e15 and e20) maternal and fetal placenta (MP and FP, respectively) of control, MH, and PI rats were dissected and frozen in liquid nitrogen. Each rat group consisted of randomly selected embryos or pups from different dams to minimize litter bias.

### 4.5. ELISA Analysis of Corticosterone Levels

Cytoplasmic fractions from the brain samples were extracted using the Nuclear and Cytoplasmic Protein Extraction Kit (78833, NEPERTM Nuclear and Cytoplasmic Extraction Reagents, Thermo Fisher Scientific, Waltham, MA, USA) and corticosterone levels were assayed using a competitive ELISA kit (AC-14F1, Xema, Moscow, Russia) according to the assay protocol, and read using a spectrophotometric plate reader (CLARIOstar PLUS, BMG Labtech, Ortenberg, Germany). The amount of corticosterone was calculated using a standard curve and expressed as pmol per mg of total protein. Here and in the other biochemical tests below, the total protein content was assessed using a PierceTM Rapid Gold BCA Protein Assay Kit (Thermo Fisher Scientific, Waltham, MA, USA) following the manufacturer’s protocol.

### 4.6. Western Blotting

To obtain total protein extracts for Western blotting, brain, MP, and FP samples were homogenized in 50 mM Tris-HCl (TBS, pH 8.0) containing 150 mM NaCl, 1% Triton X100 and a cocktail of protease and phosphatase inhibitors (SB-G2006, SB-G2007, Servicebio, Wuhan, China). Homogenates were incubated on a shaker for 30 min at +4 °C, centrifuged for 10 min at 14,000× *g*, and supernatants were collected. Samples containing equal amounts of total protein were boiled for 10 min at +70 °C with a 3× Laemmli buffer.

Proteins were separated by sodium dodecyl sulfate-polyacrylamide gel electrophoresis (SDS-PAGE) and transferred to the PVDF membranes (Thermo Fisher Scientific, Waltham, MA, USA). After blocking for 1 h in PBS containing 5% skim milk, the membranes were incubated in PBS with rabbit anti-HIF1α (1:2000, AF1009, Affinity Biosciences, USA), anti-HSD11B1 (1:2000, DF3972, Affinity Biosciences, Cincinnati, OH, USA), anti-HSD11B2 (1:2000, DF9418, Affinity Biosciences, USA), anti-GR (1:2000, AF5004, Affinity Biosciences, USA), anti-LDHA (1:2000, AF7672, Affinity Biosciences, USA), and anti-β-Tubulin (1:5000, ab179513, Abcam, Cambridge, UK) primary antibodies for 2 h at room temperature.

The membranes were then washed thrice with PBST (TBS with 0.1% Tween 20) and incubated in PBS with HRP-conjugated anti-rabbit (1:5000, E-AB-1003, Elabscience, Houston, TX, USA) secondary antibodies for 1 h at room temperature. The membranes were then washed twice with PBST. Immunoreactive protein bands were visualized using a Clarity ECL chemiluminescence kit (Bio-Rad, Hercules, CA, USA) with a ChemiScope 6000 Imaging System (Clinx Science Instruments, Shanghai, China).

Protein levels were quantified using ImageJ 1.54 software (NIH, Bethesda, MD, USA) and normalized to β-Tubulin. The results of the molecular weight testing for the antibodies used for Western blotting and full images of the Western blots are presented in the Supplementary Materials (Figures S1–S3).

### 4.7. Quantitative RT PCR

Total RNA from the brain, MP, and FP samples was isolated using the ExtractRNA Kit (BC032, Evrogen, Moscow, Russia) and purified using DNAseI (EN0521, Fermentas, Waltham, MA, USA) and the CleanRNA Standard kit (BC033, Evrogen, Moscow, Russia) according to the manufacturer’s instructions. The quality and concentration of total RNA were determined by measuring the optical density at 260 nm and 280 nm using a Nanodrop spectrophotometer (Thermo Fisher Scientific, Waltham, MA, USA). cDNA templates were synthesized from 2 μg of total RNA using the MMLV Reverse Transcription Kit (SK021, Evrogen, Moscow, Russia). Quantitative reverse transcription polymerase chain reaction (RT PCR) was carried out with qPCRmix-HS SYBR+LowROX (Evrogen, Moscow, Russia) on a SimpliAmp Thermal Cycler (Applied Biosystems, Waltham, MA, USA). Primer sequences, annealing temperatures, and fragment sizes are listed in Table 1. The expression levels of the target genes were estimated using the ΔΔCt method with normalization to *beta-tubulin* mRNA content as a reference gene.

### 4.8. Measurement of Lactate Dehydrogenase Activity

LDH activity was analyzed using a colorimetric assay (E-BC-K046-M; Elabscience, Houston, TX, USA). Dissected brain samples were washed and homogenized with PBS (0.01 M, pH 7.4) at +4 °C and centrifuged at 10,000× *g* for 10 min to isolate the cytosolic proteins. The assay procedures were conducted following the manufacturer’s protocol, and the absorbance was measured at 450 nm using a spectrophotometric microplate reader (CLARIOstar PLUS, BMG Labtech, Ortenberg, Germany). The amount of pyruvate generated during the reaction was determined by using a standard pyruvate curve. LDH activity was calculated as nmol of pyruvate generated per minute per mg of total protein.

### 4.9. Measurement of Lactate Levels

Lactate levels were analyzed using a colorimetric assay (E-BC-K044-M; Elabscience, Houston, TX, USA). Dissected brain samples were washed and homogenized with PBS (0.01 M, pH 7.4) at +4 °C and centrifuged at 10,000× *g* for 10 min to isolate the lactate-containing supernatant. The assay procedures were conducted following the manufacturer’s protocol, and the absorbance was measured at 530 nm using a spectrophotometric microplate reader (CLARIOstar PLUS, BMG Labtech, Ortenberg, Germany). The amount of lactate was quantified using a standard curve and calculated and expressed as nmol of lactate per mg of total protein.

### 4.10. Measurement of Pyruvate Levels

Pyruvate levels were analyzed using a colorimetric assay (E-BC-K130-M; Elabscience, Houston, TX, USA). Dissected brain samples were washed and homogenized with PBS (0.01 M, pH 7.4) at +4 °C and centrifuged at 10,000× *g* for 10 min to isolate the pyruvate-containing supernatant. The assay procedures were conducted following the manufacturer’s protocol, and the absorbance was measured at 505 nm using a spectrophotometric microplate reader (CLARIOstar PLUS, BMG Labtech, Ortenberg, Germany). The amount of pyruvate was quantified using a standard curve and calculated and expressed as nmol of pyruvate per mg of total protein.

### 4.11. Measurement of Glucose-6-Phosphate Dehydrogenase Activity

G6PD activity was analyzed using a colorimetric assay (E-BC-K056-M; Elabscience, TX, Houston, USA). Dissected brain samples were washed and homogenized with extraction buffer at +4 °C and centrifuged at 10,000× *g* for 10 min to isolate the cytosolic proteins. The assay procedures were conducted following the manufacturer’s protocol, and the absorbance was measured at 450 nm using a spectrophotometric microplate reader (CLARIOstar PLUS, BMG Labtech, Ortenberg, Germany). The amount of NADPH reduced during the reaction was determined by using a standard NADPH curve. G6PD activity was calculated as nmol of NADPH generated per minute per mg of total protein.

### 4.12. Measurement of NADPH Levels

The NADPH/NADP total ratio was analyzed using a colorimetric assay (E-BC-K803-M, Elabscience, Houston, TX, USA), in accordance with the manufacturer’s protocol. Dissected brain samples were homogenized with extraction buffer and centrifuged at 12,000× *g* for 10 min to isolate the NADPH/NADP+-containing supernatants. For NADPH, only detection supernatants were additionally heated at 60 °C for 30 min to decompose NADP+, cooled on ice, and spun at 10,000× *g* for 10 min to remove the precipitate. The assay procedures were conducted following the manufacturer’s protocol, and the absorbance was measured at 450 nm using a spectrophotometric microplate reader (CLARIOstar PLUS, BMG Labtech, Ortenberg, Germany). The total amount of NADPH or NADP was quantified using a standard curve and calculated as nmol per mg of total protein.

### 4.13. Measurement of Reduced Glutathione Levels

Reduced glutathione (GSHred) levels were analyzed using a colorimetric assay (E-BC-K030-M; Elabscience, Houston, TX, USA). Dissected brain samples were washed with PBS (0.01 M, pH 7.4) and homogenized in 50 mM Tris-HCl (pH 7.4) containing 150 mM NaCl, 1mM EDTA, and 1% Triton X100 at +4 °C and centrifuged at 10,000× *g* for 10 min to isolate the GSH-containing supernatant. The assay procedures were conducted following the manufacturer’s protocol, and the absorbance was measured at 405 nm using a spectrophotometric microplate reader (CLARIOstar PLUS, BMG Labtech, Ortenberg, Germany). The amount of GSHred was quantified using a standard curve, calculated, and expressed as nmol of GSHred per mg of total protein.

### 4.14. Measurement of Malonic Dialdehyde Levels

Malonic dialdehyde (MDA) levels were analyzed using a colorimetric assay (E-BC-K025-M, Elabscience, Houston, TX, USA) according to the manufacturer’s protocol. Dissected HPC samples were washed and homogenized with PBS (0.01 M, pH 7.4) at +4 °C and centrifuged at 10,000× *g* for 10 min to isolate the MDA-containing supernatant. The assay procedures were conducted following the manufacturer’s protocol, and the absorbance was measured at 532 nm using a spectrophotometric microplate reader (CLARIOstar PLUS, BMG Labtech, Ortenberg, Germany). The amount of MDA was quantified using a standard curve, calculated, and expressed as nmol of MDA per mg of total protein.

### 4.15. Statistical Analysis

All statistical analyses were conducted using R (version 4.0.2, R Foundation for Statistical Computing, Vienna, Austria) with appropriate libraries. Normality of the data was assessed using the Shapiro-Wilk test (*p* > 0.05). Parametric tests, including one-way ANOVA (with group factor for intraday comparisons or time factor for comparisons across prenatal days) and Student’s *t*-test, were applied when assumptions were met, with Tukey’s post-hoc test used for multiple comparisons. When Levene’s test indicated heterogeneity of variance, Welch’s ANOVA with Dunnett’s post-hoc test or Welch’s *t*-test were used. For non-parametric data, the Kruskal-Wallis test was used for one-way analyses, followed by Dunn’s post-hoc test, and the Mann-Whitney U test was employed for paired comparisons. Results are presented as mean ± standard error of the mean (SEM) for parametric data, and as box-and-whisker plots for non-parametric data.

## Figures and Tables

**Table 1:** Primers used for quantitative RT PCR.

Gene	Primers Sequences (5′–3′)	Annealing T (°C)	Product Size (bp)
*beta-tubulin*	Forward TAGAGGAGATGCTACTTA Reverse AATGGTGATAATACTGTTAA	58	147
*dusp1*	Forward ACCAGTATTAACCATTCC Reverse TTCGTTCTTCTATGAGTAG	57	115
*fkbp5*	Forward ATCTGCCACTTATTATGTAA Reverse AGTCAAGGAGTTCAATCT	57	106
*g6pd*	Forward AAGATGATGACCAAGAAG Reverse TTGTATCTGTTGCCATAG	56	80
*glut1*	Forward AATATGTGGAGCAACTGT Reverse TAGGTGAAGATGAAGAAGAG	56	79
*nr3c1*	Forward ATCATACAGACAATCAAG Reverse TACTCTTCATAGGATACC	58	156
*hk1*	Forward CTGGACTGTGGAATCTTG Reverse AGTAAGGAGGCTACATCAT	56	80
*hif1α*	Forward CCATTCCTCATCCATCAA Reverse CCATCAACTCAGTAATCCT	56	114
*hsd11b1*	Forward CCTCTGATTGCTTCCTAC Reverse TTGGTCATCAAGTGTTCT	57	83
*hsd11b2*	Forward CCTATGGTGAAGACTACA Reverse AATGATGGCATCTACAAC	57	98
*ldha*	Forward CGAGAGCATAATGAAGAAC Reverse TCCTTGATTCCATAGAGAC	56	75
*mct4*	Forward CCTATATTGCCAATCCTCCAT Reverse TGTCTATCTCTGCCTTCTCA	57	75
*pdk1*	Forward TACTCAACCAGCACTCTT Reverse GCATTCTCATAGCCATCTT	57	121
*pfkb3*	Forward ATCTATGAGTGAGTGTCT Reverse ATGGTAATAGTGAGTATGC	56	84
*zbtb16*	Forward GATGAAGACATACGGATG Reverse TGAATGAGCCAGTAAATG	57	82

## Data Availability

The data supporting the findings of this study are present in the paper and/or the Supplementary Materials.

## Supplementary Materials

The following supporting information can be downloaded at: https://www.mdpi.com/article/10.3390/ijms252413342/s1.
