# Supplementary material for: Comparative Analysis of the Effects of Maternal Hypoxia and Placental Ischemia on HIF1-Dependent Metabolism and the Glucocorticoid System in the Embryonic and Newborn Rat Brain
Source: Int J Mol Sci. 2024 Dec 12;25(24):13342. doi: 10.3390/ijms252413342 (PMC11727977; doi:10.3390/ijms252413342)
Supplement: Supplementary file 1 [file ijms-25-13342-s001.zip › ijms-3293662-supplementary.pdf]

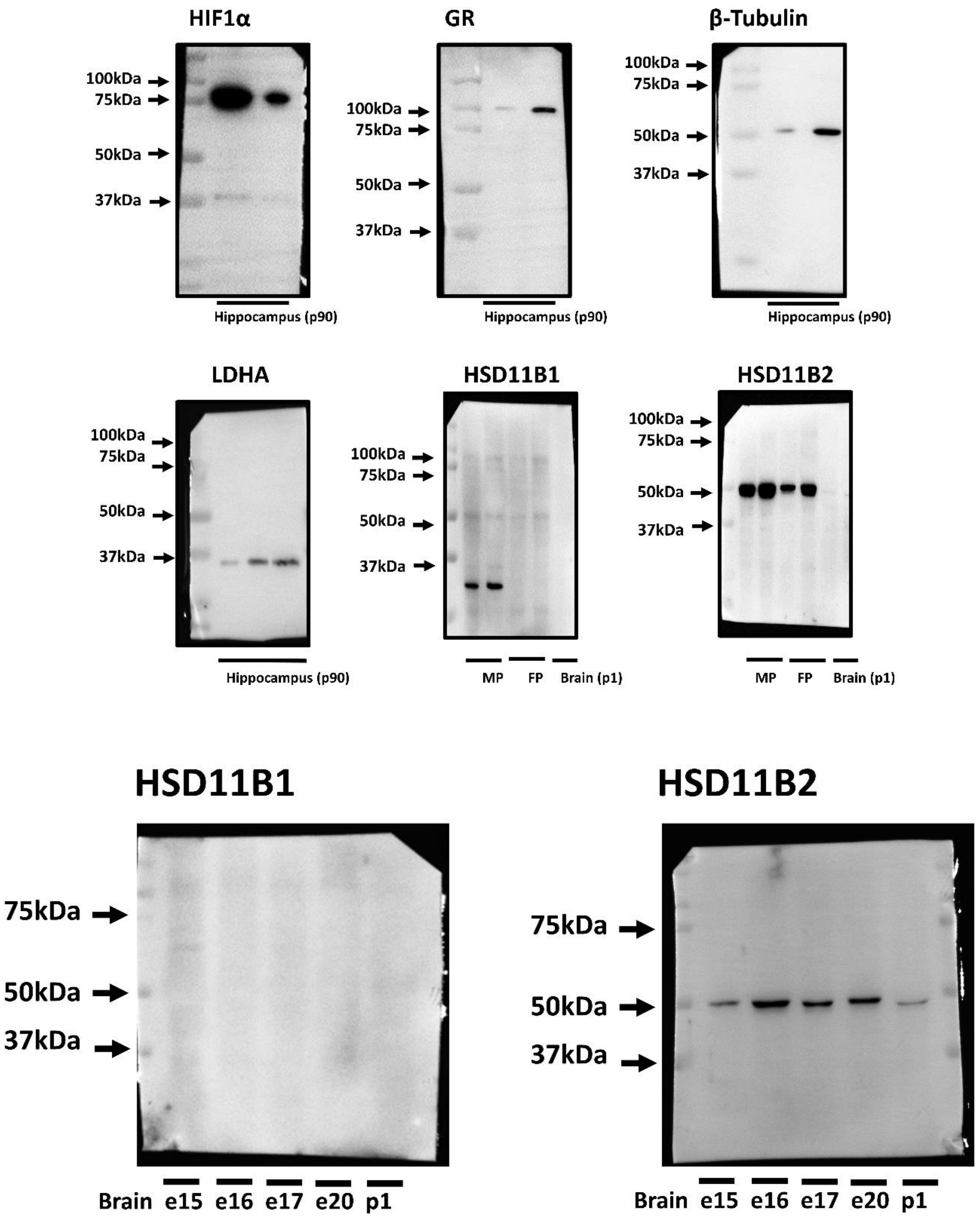

Fig. S1. Molecular weight testing for the antibodies used for Western blotting.

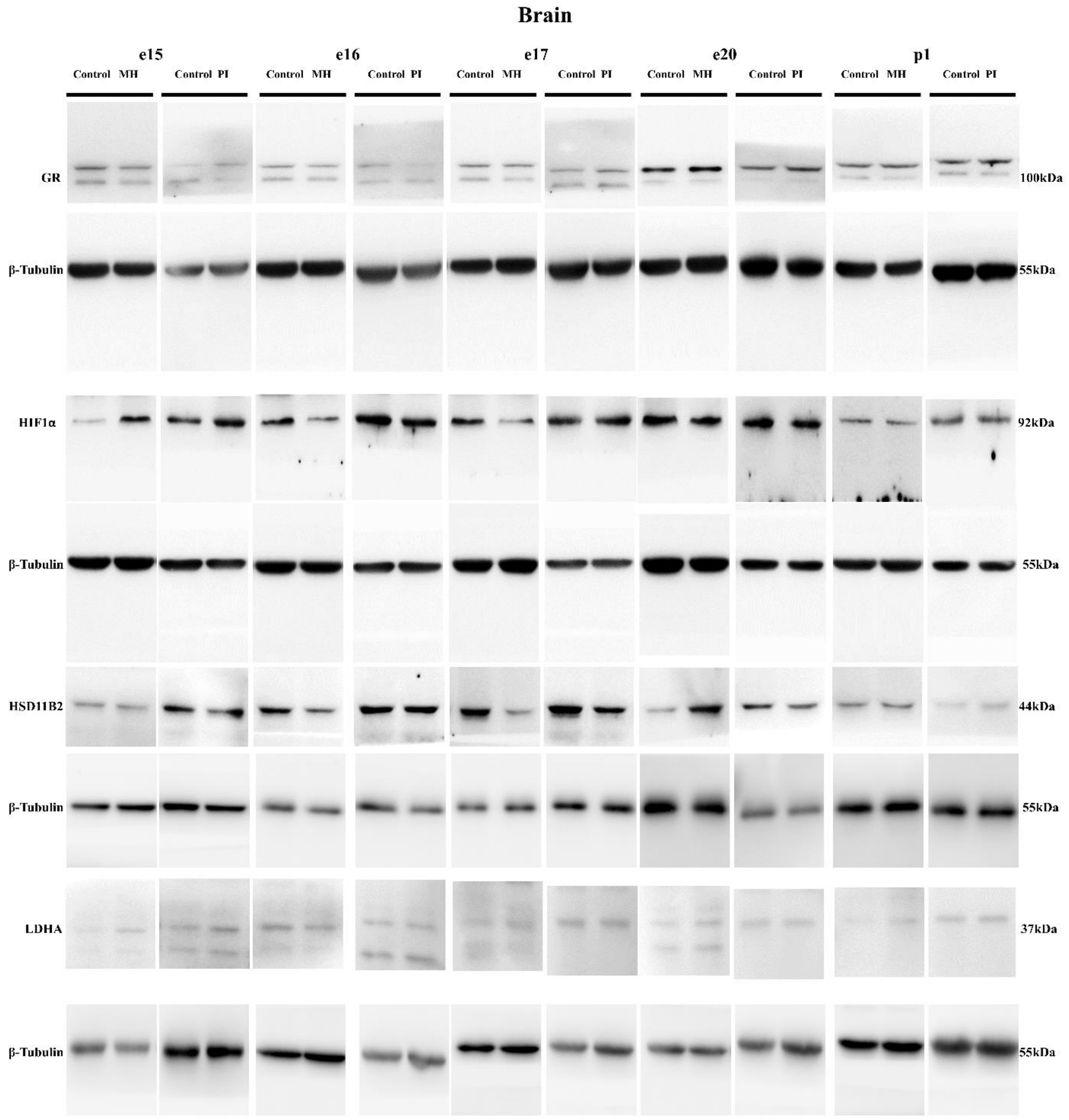

Fig. S2. Full images of the Western blot results (brains).

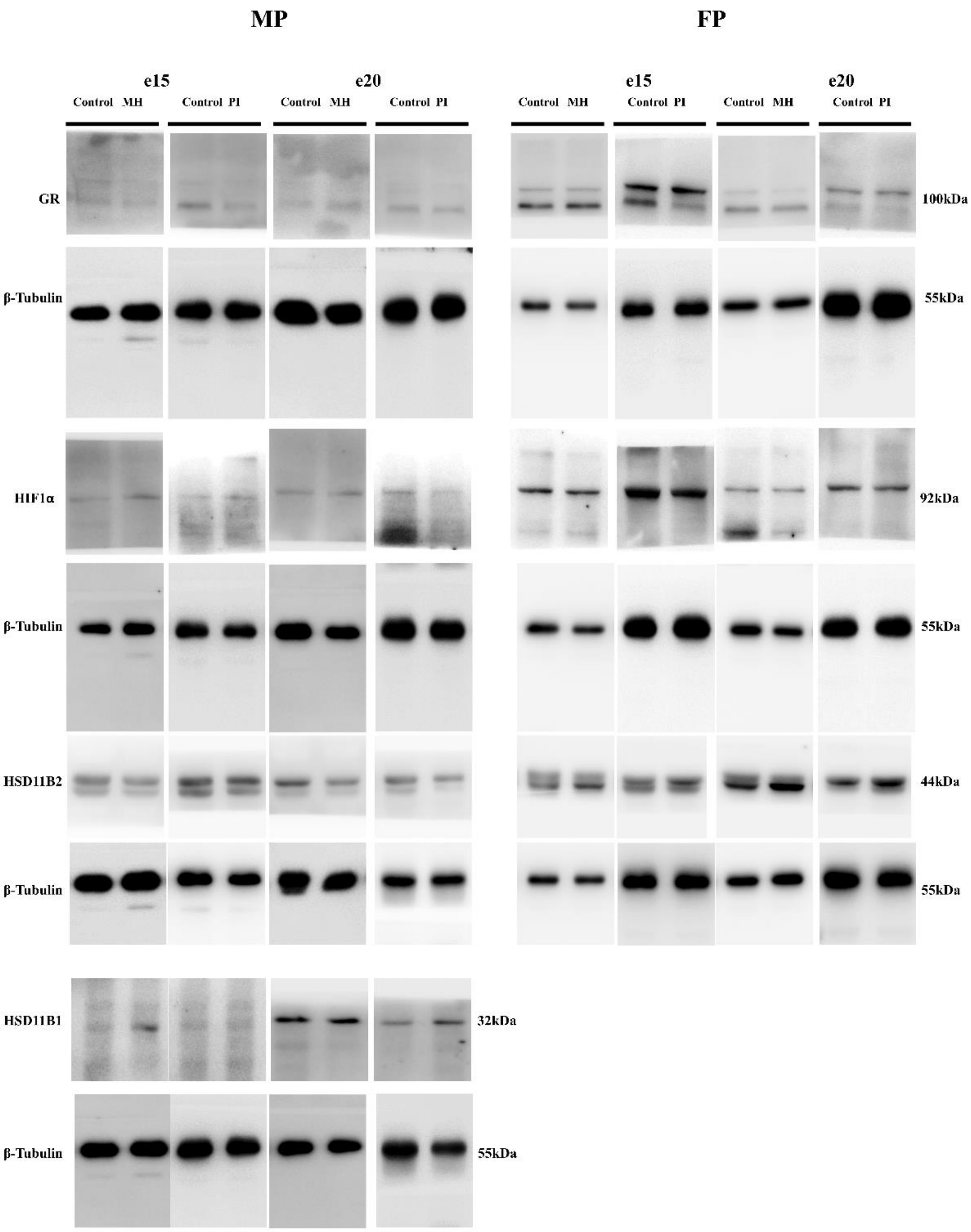

Fig. S3. Full images of the Western blot results (MP and FP).
